# Impact of Mandatory Clinical Ethics Consultation for Neurocritically Ill Patients

**DOI:** 10.1007/s11673-025-10512-w

**Published:** 2026-05-28

**Authors:** Yen-Ko Lin, Chao-Wen Chen, Yung-Sung Yeh, Chia-Ju Lin, Yu-Wen Huang, Yu-Chih Lin, Chau-Chyun Sheu

**Affiliations:** 1 Division of Trauma and Surgical Critical Care, Department of Surgery, Kaohsiung Medical University Hospital, Kaohsiung Medical University, No.100, Tzyou 1 st Rd., Sanmin District Kaohsiung City, 807378 Taiwan; 2 Department of Emergency Medicine, Kaohsiung Medical University Hospital, Kaohsiung Medical University, No.100, Tzyou 1 st Rd., Sanmin District Kaohsiung City, 807378 Taiwan; 3 Department of Medical Humanities and Education, College of Medicine, Kaohsiung Medical University, No.100, Shiquan 1 st Rd., Sanmin District Kaohsiung City, 807378 Taiwan; 4 Center for Medical Education and Humanizing Health Professional Education, Kaohsiung Medical University, No.100, Shiquan 1 st Rd., Sanmin District Kaohsiung City, 807378 Taiwan; 5 Department of Emergency Medicine, College of Medicine, Kaohsiung Medical University, No.100, Shiquan 1 st Rd., Sanmin District Kaohsiung City, 807378 Taiwan; 6 College of Nursing, Kaohsiung Medical University, No. 100, Shiquan 1 st Rd., Sanmin District Kaohsiung City, 807378 Taiwan; 7 Department of Nursing, Kaohsiung Medical University Hospital, Kaohsiung Medical University, No. 100, Tzyou 1 st Rd., Sanmin District Kaohsiung City, 807378 Taiwan; 8 Division of Rheumatology, Department of Internal Medicine, College of Medicine, Kaohsiung Medical University Hospital, Kaohsiung Medical University, No. 100, Tzyou 1 st Rd., Sanmin District Kaohsiung City, 807378 Taiwan; 9 Department of Internal Medicine, College of Medicine, Kaohsiung Medical University, No. 100, Shiquan 1 st Rd., Sanmin District Kaohsiung City, 807378 Taiwan; 10 Division of Pulmonary and Critical Care Medicine, Department of Internal Medicine, Kaohsiung Medical University Hospital, Kaohsiung Medical University, No. 100, Tzyou 1 st Rd., Sanmin District Kaohsiung City, 807378 Taiwan

**Keywords:** Mandatory clinical ethics consultation, Intensive care unit, Withdrawal of life-sustaining treatment, End of life, Neurocritical care

## Abstract

This study aimed to investigate and compare the longitudinal impacts of mandatory clinical ethics consultation (CEC) on resource use and ethical conflicts among critically ill neurologic and non-neurologic patients. It also sought to identify relevant predictive factors and determine family member satisfaction with CEC. This combined retrospective and prospective cohort study was conducted at a tertiary academic medical centre. During the eight-year study period, 1,150 adult patients participated in this study (437 neurological and 713 non-neurological). In both groups, the ICU length of stay and days of ventilator use significantly decreased each year. Secular trends in the incidence rates of ethical conflicts significantly decreased each year in neurological patients. Neurological patients exhibited a greater decrease in resource usage and significantly higher incidence rates of ethical conflicts than non-neurological patients. Family members of both groups expressed high levels of satisfaction and agreed that CEC was beneficial. In the local healthcare context, the implementation of CEC policies may reduce resource usage, ethical conflicts, and improve the knowledge base of CEC and clinical teams, helping them in approaching the distinct issues raised by various patient characteristics. However, these findings should be interpreted with caution as their applicability to other healthcare systems may vary.

**Supplementary Information:**

The online version contains supplementary material available at https://doi.org/10.1007/s11673-025-10512-w.

## Introduction

Clinical ethics consultation (CEC) has been implemented in various hospitals because it is recognized as an effective approach for enhancing communication and addressing value-laden conflicts in healthcare contexts (Fox, Myers, and Pearlman 2007; Tarzian and ASBH Core Competencies Update Task Force 1,2013; Filippini et al. 2022; Fox et al. 2022). Healthcare providers often encounter ethical dilemmas in their daily practice, requiring thoughtful reflection and decision-making. Some healthcare providers do not possess the requisite training and knowledge to effectively address such challenging circumstances (Prien and Van Aken 1999; Tapper 2013). CEC demonstrates significant potential in mitigating unnecessary and undesired treatments, addressing ethical dilemmas, and providing essential support to healthcare providers and family members involved in end-of-life care (Schneiderman, Gilmer, and Teetzel 2000; Schneiderman et al. 2003, Schneiderman 2005, 2006). CEC is critical in the intensive care unit (ICU), particularly concerning end-of-life care. Consequently, institutions should prioritize implementing CEC policies so as to effectively address the aforementioned issues (Filippini et al. 2022).

Mandatory CEC should be initiated in accordance with institutional policies that delineate the specific circumstances which necessitate their activation. Numerous studies have advocated mandating CEC under particular conditions to facilitate informed treatment decision-making, mitigate individual biases, enhance communication, and ensure the provision of high-quality care (Romano et al. 2009; Neal et al. 2020). The implementation of mandatory CEC has yielded positive outcomes, suggesting that it may enhance the working relationship between ethics consultants and physicians. Furthermore, it may facilitate the learning process for physicians and increase awareness of ways to offer ethical assistance among both physicians and hospital staff (Romano et al. 2009).

Most fatalities in the ICU occur after the withdrawal of life-sustaining treatments (Prendergast, Claessens, and Luce 1998). The withholding or withdrawal of life-sustaining treatments serves as the principal rationale for initiating CEC within ICUs (Wasson et al. 2015). When a patients’ condition significantly deteriorates, the likelihood of survival diminishes considerably. Decisions to withhold or withdraw treatment can lead to ethical dilemmas and emotional distress for families and healthcare providers in ICUs. In this context, the timing of critical decisions often presents additional challenges (Romano et al. 2009; Choi, Ho, and Lin 2024a, 2024b; Cortegiani, Ippolito, and Mercadante 2024; Palmryd et al. 2025).

Neurological diseases are the leading causes of disability and the second leading cause of death worldwide (Feigin et al. 2019). Owing to their special characteristics, the treatment of neurologic patients may raise unique ethical issues. With this in mind, CEC is feasible for consensus-finding not only in conflicts but also in situations of therapeutic uncertainty in neurology (Ilse et al. 2021). CEC may be of particular importance in neurology for several reasons. The symptoms of patients with neurological disorders are often complex and diverse (Elmer et al. 2016). The characteristic courses of such diseases are often long and frequently complicated by behavioural changes, cognitive impairment, and progressive speech and language disorders. Critically ill neurological patients have impaired cognitive function, and most are incapable of expressing their wishes and providing consent. These factors may have contributed to the increased use of CEC in neurology.

To date, no research has yet reported on the comprehensive and long-term effects of the implementation of a mandatory CEC policy, nor have relevant comparisons been made between neurologic and non-neurologic patients concerning them. This study aimed to investigate and compare the longitudinal impacts of mandatory CEC on resource usage and ethical conflicts in the treatment of critically ill neurological and non-neurological patients, as well as their predictive factors.

## Methods

### Data Processing

This study presents a combined retrospective and prospective cohort analysis that was conducted at Kaohsiung Medical University Hospital, a tertiary academic medical centre—with a capacity of 1,300 beds—situated in Southern Taiwan. The study included patients aged eighteen years and older who were admitted to adult ICUs and underwent mandatory CEC between January 1, 2013, and December 31, 2020. Data including admission notes, progress notes, discharge notes, and records from family meetings and ethics consultations were obtained using the hospital’s computer system. The data collected included various factors, such as age, sex, current marital status, admission year, surrogate decision-maker, expression of wishes, advance directives, episodes of cardiac arrest, and the administration of inotropes or vasopressors. We also recorded whether the patients died in the hospital, along with their Glasgow Coma Scale (GCS) scores. Furthermore, the data included the ICU length of stay (LOS), total hospital LOS, days of ventilator use, duration from ICU admission to ethics consultation, time from ethics consultation to withdrawal of life-sustaining treatment, and ethical contexts. As part of the study, this prospectively collected data was subject to retrospective analysis.

The ethical contexts and categorization of ethical issues were deliberated and established by consensus of the consultation team, and these determinations were recorded in the CEC notes after completing the CEC process. Upon activation of the CEC, the consultation team performed a comprehensive assessment by visiting the patient and engaging in dialogue with the clinical team. They meticulously reviewed all relevant documentation and played a key role in organizing meetings with patient families. These structured meetings focused on the ethical considerations and the process surrounding the withdrawal of life-sustaining treatments and were facilitated by the consultation team. The meetings brought together members of the CEC, the clinical team, and the patient’s family, fostering a collaborative discourse on the patient’s care plan. Ethical contexts were thoroughly discussed and established through consensus among the members of the consultation team, based on defined criteria. These discussions were recorded in the CEC notes at the conclusion of the CEC process. Two independent investigators, LYK and HYW, were involved in the consultation process, during which they assessed the records and compiled the relevant data for further analysis. The data on family member satisfaction were collected between 2020 and 2023. The study protocol was approved by the Institutional Review Board of Kaohsiung Medical University Hospital. The Institutional Review Board (IRB) number is KMUH-IRB-20130379.

### Mandatory CEC Policy

In 2013, Taiwan formally enacted amendments to the Hospice Palliative Care Act, resulting in a revised framework to enhance the provision of palliative care services (Ministry of Health and Welfare 2021). The revised amendments modified the procedures for patients undergoing the withholding or withdrawal of life-sustaining treatments. Two specialists were required to evaluate whether a patient’s condition was terminal. In instances where no advance directive had been established by the patient, a family member was usually designated as a surrogate decision-maker under related healthcare legislation. In some cases, the surrogate may also be a formally appointed healthcare agent if previously designated by the patient. In 2013, Kaohsiung Medical University Hospital introduced an institutional mandatory CEC policy to guide the development of internal regulations and improve the quality of end-of-life care. This policy mandated that CEC be conducted for all patients undergoing the withdrawal of life-sustaining treatments. Consequently, CEC is mandatory whenever patients, family members, or healthcare providers request cessation of such treatments.

### CEC Teams and Processes

CEC services are one of the principal functions of the ethics committee within Kaohsiung Medical University Hospital. The CEC team included a physician—who also served as team leader—, a nurse practitioner specializing in hospice and palliative care, a social worker, and a clinical psychologist. The team leader was a trauma surgeon with over twenty years of experience in emergency and critical care and a graduate degree in bioethics. The primary responsibility of the team leader was to identify ethical dilemmas, mediate conflicts related to ethics, and, when necessary, serve as a second specialist in assessing the terminal conditions of patients. Nursing practitioners with expertise in hospice and palliative care offer professional guidance on end-of-life care. Social workers assist patients and their families in obtaining social and financial resources and facilitate the understanding of the relationships between patients and their surrogate decision-makers. The clinical psychologist provided bereavement counselling when necessary. The consultation team continued to engage with the patients throughout their care until they were discharged or expired.

Structured family meetings were conducted to facilitate open communication on patient care. The CEC process provided an avenue through which both the family members and clinical team could express their appreciation for the dedication and efforts of the other. The CEC also elucidated the fundamental principles governing the withdrawal of life-sustaining treatment while outlining the pertinent regulations and laws associated with this process. The CEC team emphasized that the withdrawal of life-sustaining treatment is not an act of abandonment but rather a way to alleviate the patient’s burden and provide comfortable care in accordance with the patient’s wishes. In our CEC framework, the patient’s values, preferences, and previously expressed wishes are given the highest ethical and legal priority, particularly when considering withdrawal of life-sustaining treatment. When patients retain decision-making capacity, their autonomous choices are directly respected. In cases where the patient lacks capacity, advance directives are followed. In the absence of such directives, surrogates are asked to engage in substituted judgment based on the patient’s known values and beliefs. The CEC team facilitated these discussions to ensure that decisions remained aligned with the patient’s best interests and presumed wishes, and to avoid actions that may have caused undue suffering or contradicted the patient’s values. If consensus was established, the consultation team articulated the process involved in withdrawing life-sustaining treatment. Family members were strongly encouraged to accompany the patients throughout the withdrawal process. Additionally, suitable medications were administered to effectively alleviate any pain or suffering experienced by the patients. The clinical and consultation teams honoured the timing selected by the family members for the withdrawal process. This consideration provided family members the time necessary to invite other relatives to extend their final goodbyes and make appropriate arrangements following the patient’s death. The CEC members actively participated in and facilitated the entire process of withdrawing life-sustaining treatment by offering ethical guidance, clarifying the consensus among stakeholders, and supporting emotionally complex family discussions. The presence of CEC members helped ensure alignment between the patient’s values, legal standards, and institutional policies. Once consensus was achieved and the decision to withdraw treatment was made, the clinical team carried out the relevant medical procedures, such as titrating medications, stopping mechanical ventilation, or discontinuing dialysis. The CEC team remained available during and after this stage to provide emotional support, ensure ethical consistency, and assist in post-withdrawal communication if needed. If the patient survived after withdrawal, a nursing practitioner from the CEC team coordinated the transfer to the hospice ward and assumed responsibility for patient care.

### Outcome Measurement

#### Length of stay and Ethical Contexts

The outcome measures evaluated in this study included the ICU LOS, total hospital LOS, days of ventilator use, interval from ICU admission to ethics consultation, and period from ethics consultation to withdrawal of life-sustaining treatment. These metrics were analysed and compared between patients with and without neurological conditions. Furthermore, the ethical contexts were systematically analysed and compared. This analysis encompassed conflicts regarding the determination of terminally ill conditions, conflicts between family members, conflicts between the family members and clinical team, and conflicts between clinical team members. “Conflicts regarding the determination of terminally ill conditions” were defined as any uncertainty, disagreement, or dilemma on the same. “Ethical conflicts” arose when families and clinical teams disagreed on prognosis or whether patients met terminal criteria, often requiring ethics consultation to bridge differing perspectives on treatment futility and end-of-life decisions. Similarly, “conflicts that arise among family members, between family members and the clinical team, and conflicts between clinical team members” are defined as any instances of uncertainty, disagreement, or dilemmas that occur within these specific interactions.

#### Family Member Satisfaction

A structured interview was conducted with surrogates by the research assistant, either in person or via telephone, within one to two weeks after the patient’s death. A panel of experts was invited to participate in the development of the questionnaire used for the interviews. This panel included twelve physicians and five nurses/nurse practitioners who each had more than one year of experience of end-of-life and critical care, one judge, two lawyers, three experts with bioethics expertise, and five social workers. Eighteen questions were developed and distributed to the panel of experts. The experts were asked to rate each item on a five-point scale from “strong agreement” to “strong disagreement.” The rating results were analysed, and the ten top-ranked questions were selected. The responses to the questionnaire demonstrated a high level of consistency. Informed consent to participate in the interviews was obtained during family meetings, and all individuals interviewed had engaged in the ethics consultation process. Participants were selected through convenience sampling and were required to complete a structured questionnaire formatted on a Likert scale. The questionnaire focused on critical areas for family members, including satisfaction with the clinical team, the consultation team, and the family meeting; opinions regarding whether the consultation team helped in making medical decisions; whether the patient suffered during the withdrawal process; whether all concerns had been resolved; whether their values or perspectives were respected; whether they will recommend CEC to others; and whether CEC services should be provided for end-of-life care.

### Statistical Analyses

All data collected from the participants were recorded using assigned participant numbers, omitting any specific identifying information to safeguard the participants’ privacy and ensure confidentiality. The analysis was conducted to quantify the secular changes in LOS, defined as long-term trends over the study period, potentially reflecting broader shifts in clinical practice through the application of the Cuzick nonparametric test (Cuzick 1985). Similarly, we conducted an analysis to quantify the secular changes in ethical contexts using the Cochran-Armitage test (Cochran 1954; Armitage 1955). Student t-tests was used to evaluate the associations between numerical and categorical clinical variables of interest as well as their relationships with the ICU LOS, total LOS, and duration of ventilator use. Fisher’s exact test was used to analyse the binary, ordinal, and categorical variables. Multiple linear regression models were used to evaluate the ICU LOS, total LOS, and duration of ventilator use, incorporating predefined covariates. Furthermore, multivariate logistic regression models were applied to determine the incidence rate in ethical contexts with the inclusion of predefined covariates. Likelihood ratio tests were conducted to assess the multivariable models. A significance threshold of *p* < 0.05 was established for the variables under consideration. Additionally, 95 per cent confidence intervals were calculated to provide further insight into the data. All data analyses were performed using Stata version 18.0 (StataCorp LP, College Station, TX, USA).

## Results

A total of 1,150 adult patients were included in this study between 2013 and 2020. A total of 437 patients were neurologic and 713 were non-neurologic. A comparison of the sociodemographic and clinical characteristics between neurological and non-neurological diseases is presented in Supplementary Table 1. While the non-neurologic patients had an average age of 68.36 years, neurologic patients were significantly younger, with an average age of 63.52 years. The frequency of ethics consultations increased annually in both groups. The majority of surrogates were offspring in both groups. Only sixteen neurologic patients (3.7 per cent) and thirty-four non-neurologic patients (4.8 per cent) had advanced directives. However, 78 neurologic patients (17.9 per cent) and 176 non-neurologic patients (24.7 per cent) expressed their wishes for end-of-life care. More non-neurological patients experienced episodes of cardiac arrest and received inotropes or vasopressors. After the withdrawal of life-sustaining treatments, 328 neurologic patients (75.1 per cent) and 674 non-neurologic patients (94.5 per cent) died in the hospital. Consciousness levels were significantly poorer in neurological patients, and the mean GCS score was 4.60.

Secular trends and comparisons of neurological and non-neurological patients for the ICU LOS, total LOS, days of ventilator use, days from ICU admission to ethics consultation, and days from ethics consultation to the withdrawal of life-sustaining treatments are presented in Supplementary Table 2. For neurological patients, the ICU LOS, total LOS, days of ventilator use, number of days from ICU admission to ethics consultation, and number of days from ethics consultation to withdrawal of life-sustaining treatments significantly decreased. In particular, the means decreased by 8.7 per cent for ICU LOS (from 18.17 between 2013–2014 to 16.59 between 2018–2020), 23.4 per cent for total LOS (from 30.07 between 2013–2014 to 23.02 between 2018–2020), 31.8 per cent for days of ventilator use (from 22.04 between 2013–2014 to 15.04 between 2018–2020), 29 per cent for days from ICU admission to ethics consultation (from 17.02 between 2013–2014 to 12.08 between 2018–2020), and 48.2 per cent for days from ethics consultation to withdrawal of life-sustaining treatments (from 4.98 in 2013–2014 to 2.58 in 2018–2020). For non-neurologic patients, the number of days of ventilator use and ICU admission for ethics consultation significantly decreased. More specifically, the means decreased by 14.5 per cent for ICU LOS (from 19.53 between 2013–2014 to 16.70 between 2018–2020), 17.1 per cent for days of ventilator use (from 19.53 between 2013–2014 to 16.19 between 2018–2020), 17.0 per cent for days from ICU admission to ethics consultation (from 16.92 between 2013–2014 to 14.05 between 2018–2020), and 43.3 per cent for days from ethics consultation to withdrawal of life-sustaining treatments (from 3.63 between 2013–2014 to 2.06 between 2018–2020).

For all years studied, there were significantly more days from ethics consultation to the withdrawal of life-sustaining treatment for neurologic patients than for non-neurologic patients. Between 2015–2017, there were significantly longer ICU LOS, days of ventilator use, and days of ethics consultation for the withdrawal of life-sustaining treatment for neurologic patients than for non-neurologic patients. Between 2018–2020, there were significantly more days from ICU admission to ethics consultation for neurologic patients than for non-neurologic patients.

Subgroup analyses of the ICU LOS, total LOS, and days of ventilator use between neurologic and non-neurologic patients are presented in Supplementary Table 3. Neurologic patients whose GCS scores were greater than 4 had longer ICU LOS, total LOS, and days of ventilator use than non-neurologic patients.

Supplementary Table 4 presents the multiple linear regression models for the ICU LOS, total LOS, and number of days of ventilator use. Patients with advanced cancer and GCS scores significantly affected the ICU LOS for neurologic patients, with coefficients of −3.570 and 1.567 respectively. Patients with advanced cancer and those who had experienced an episode of cardiac arrest significantly affected the ICU LOS for non-neurological patients, with coefficients of −2.055 and −4.289 respectively. Age and GCS score significantly affected the total LOS for neurologic patients, with coefficients of −0.155 and 3.389 respectively. Patients who experienced an episode of cardiac arrest significantly affected the total LOS for non-neurological patients, with a coefficient of −9.530. GCS score significantly affecting the duration of ventilator use in neurological patients, with a coefficient of 1.882. Patients who experienced episodes of cardiac arrest and patients who received inotropes or vasopressors significantly affected the days of ventilator use for non-neurologic patients, with coefficients of −3.538 and −2.779 respectively.

Supplementary Table 5 lists the secular trends and a comparison of incidence rates for ethical contexts between neurologic and non-neurologic patients. The incidence rate for conflicts regarding the determination of a terminally ill condition significantly decreased in neurologic patients, from 29.6 per cent between 2013–2014 to 12.9 per cent between 2018–2020. The incidence rate for conflicts regarding the determination of a terminally ill condition decreased in non-neurologic patients from 25.3 per cent between 2013–2014 to 11.8 per cent between 2018–2020; however, this was not statistically significant. The incidence rate for conflicts between family members and clinical teams significantly decreased in neurologic patients, from 59.3 per cent between 2013–2014 to 40.1 per cent between 2018–2020. The incidence rate for conflict between family members and clinical teams significantly decreased in non-neurologic patients, from 44.6 per cent between 2013–2014 to 30.6 per cent between 2018–2020. The incidence rate for conflicts between clinical team members significantly decreased in neurologic patients, from 20.4 per cent between 2013–2014 to 10.9 per cent between 2018–2020. The incidence rate for conflicts between clinical team members significantly decreased in non-neurologic patients, from 15.7 per cent between 2013–2014 to 7.4 per cent between 2018–2020.

The incidence rates for all years were significantly higher for neurologic patients than for non-neurologic patients for conflicts between family members and clinical teams and conflicts between clinical team members. The incidence rate of conflicts regarding the determination of a terminally ill condition, conflicts between family members and clinical teams, and conflicts between clinical team members were significantly higher in neurologic patients than in non-neurologic patients from 2015 to 2017. The incidence rate of conflict between family members and the clinical team was significantly higher in neurologic patients than in non-neurologic patients from 2018–2020.

A subgroup analysis of the incidence rate for ethical contexts is shown in Supplementary Table 6. For conflicts regarding the determination of a terminally ill condition, male patients and patients with GCS scores greater than 4 had higher incidence rates among neurologic patients than among non-neurologic patients. For conflicts between family members and clinical teams, except for patients receiving inotropes or vasopressors, all subgroups of age, sex, current marital status, advanced cancer, cardiac arrest, and GCS scores had significantly higher incidence rates in neurologic patients than in non-neurologic patients. Regarding conflicts between clinical team members, patients aged less than 68 years, male patients, unmarried patients, patients without advanced cancer, patients not experiencing an episode of cardiac arrest, patients receiving inotropes or vasopressors, and patients whose GCS score was greater than 4 had significantly higher incidence rates in neurologic patients than in non-neurologic patients.

The multivariate logistic regression models of the incidence rates for ethical contexts, controlling for predefined covariates, are presented in Supplementary Table 7. For conflicts regarding the determination of a terminally ill condition, the adjusted odds ratio suggested that GCS score increased the incidence rate, with an adjusted odds ratio of 1.387 (95 per cent confidence interval [CI]: 1.216–1.583) for neurologic patients. For conflicts between family members, the adjusted odds ratio suggested that current marital status decreased and GCS score increased the incidence rate, with adjusted odds ratios of 0.618 (95 per cent CI: 0.410–0.933) and 1.225 (95 per cent CI: 1.093–1.373) for neurologic patients respectively. Concerning non-neurologic patients, the adjusted odds ratios suggested that age, sex, and current marital status decreased while the GCS score increased the incidence rate, with adjusted odds ratios of 0.988 (95 per cent CI: 0.977–0.999), 0.661 (95 per cent CI: 0.482–0.906), 0.596 (95 per cent CI: 0.432–0.822), and 1.128 (95 per cent CI: 1.060–1.201) respectively. For conflicts between family members and clinical teams, the adjusted odds ratio suggested that current marital status decreased and GCS score increased the incidence rate, with adjusted odds ratios of 0.665 (95 per cent CI: 0.446–0.992) and 1.113 (95 per cent CI: 1.003–1.236) for neurologic patients respectively. Concerning non-neurologic patients, the adjusted odds ratio suggested that current marital status and advanced cancer decreased and GCS score increased the incidence rate, with adjusted odds ratios of 0.707 (95 per cent CI: 0.510–0.980), 0.711 (95 per cent CI: 0.510–0.991), and 1.132 (95 per cent CI: 1.064–1.204) respectively. For conflicts between the clinical team members, the adjusted odds ratio suggested that age decreased and GCS score increased the incidence rate, with adjusted odds ratios of 0.979 (95 per cent CI: 0.964–0.995) and 1.245 (95 per cent CI: 1.097–1.414) for neurologic patients respectively. Concerning non-neurologic patients, the adjusted odds ratio indicated that receiving inotropes or vasopressors decreased and GCS score increased the incidence rate, with adjusted odds ratios of 0.505 (95 per cent CI: 0.276–0.925) and 1.139 (95 per cent CI: 1.038–1.249) respectively.

All invited participants agreed to participate in the interview. However, five participants expressed a desire not to be contacted again due to experiencing a depressed mood and feelings overwhelmed by grief. Supplementary Table 8 provides a comparative analysis of the baseline characteristics of family members during follow-up interviews conducted after the withdrawal of life-sustaining treatment in both neurological and non-neurological patient cohorts. Supplementary Table 9 examines the feedback from family members regarding CEC services. Family members from both groups expressed a high degree of satisfaction with the clinical team and the CEC team. A significant majority of family members indicated that they believed CEC facilitated the medical decision-making process, that their concerns were addressed appropriately, that their values were respected, and that they would recommend CEC to other patients or family members in the context of end-of-life care. Furthermore, they unanimously affirmed the necessity of providing CEC during end-of-life care.

## Discussion

Our study has revealed the results of the implementation of the mandatory CEC policy, compared the differences and associated factors, and examined the predictive factors for resource usage and ethical contexts in neurologic and non-neurologic patients. The results indicated that secular trends in resource usage and ethical contexts decreased in both neurologic and non-neurologic patients after the implementation of mandatory CEC. Neurologic patients exhibited a higher decrease in resource usage than non-neurologic patients. Neurologic patients had significantly higher incidence rates of ethical conflict than non-neurologic patients. Moreover, factors predicting resource usage and incidence rates of ethical conflicts in neurologic and non-neurologic patients were identified. To the best of our knowledge, this is the first comprehensive and longitudinal study to investigate and compare the impacts of a mandatory CEC policy on resource usage and ethical conflicts in neurologic and non-neurologic patients.

The implementation of the mandatory CEC policy essentially reduced resource utilization and ethical conflicts for both neurologic and non-neurologic patients, with a pronounced impact observed among neurologic patients. We believe that the mandatory CEC policy, along with our consultation model, played a pivotal role in this improvement. Our CEC team was multidisciplinary, comprised of members with specialized expertise in clinical ethics, hospice and palliative care, social work, and clinical psychology. Under the framework of the mandatory policy, the CEC process heightens the awareness of clinical team members regarding potential ethical dilemmas and the assistance available to address them (Romano et al. 2009). Furthermore, CEC creates a valuable platform for stakeholders to engage in meaningful communication, fostering an understanding of diverse opinions and perspectives. This forum not only facilitates negotiations but also aids in achieving consensus. CEC successfully increased engagement and provided essential education to the clinical team through individualized discussions with clinical team members on a case-by-case basis. Moreover, it facilitated effective communication between family members and the clinical team, as well as among members of the clinical team, which in turn contributed to addressing ethical concerns and enhancing the quality of end-of-life care.

Furthermore, our study adopted an appropriate approach to ethical consultation, one which laid emphasis upon the identification and analysis of any value uncertainties while facilitating and building to an ethical resolution (Tarzian and ASBH Core Competencies Update Task Force 1,2013). Several authors have emphasized that some of the important functions and outcomes of CEC are to facilitate understanding of the situation, explore the perspectives of different parties, and improve communication (Au et al. 2018). Despite these common functions, it is worth noting that concrete approaches to the CEC process and outcome evaluation may differ between countries and cultures.

Our CEC team is comprised of a variety of specialists, and our consultation model is unique. In particular, our CEC model combined the advantages of ethics and palliative consultation. Withholding or withdrawal of life-sustaining treatments is the most important reason to trigger CEC in intensive care settings (Wasson et al. 2015). Our results revealed that many ethical issues were raised between family members and clinical team members. The hospital staff and family members raised inquiries concerning the ethical implications and appropriateness of withdrawing life-sustaining treatments, as well as the relevant laws and regulations governing this process. Our CEC team members actively conducted visits to the ICU to promote the policy and discuss the process of withdrawing life-sustaining treatments. Moreover, the CEC team proactively approached patients who requested hospice consultation to assist the clinical team in resolving potential problems and ethical concerns, even before the CEC was formally requested. As such, our efforts may contribute to the successful development of the CEC model and consultation policy in addition to showing promising results. However, it should be emphasized that CEC models and consultation policies might develop differently in different countries and cultures, particularly as a consequence of having to take local laws into account. For instance, two specialists were required for determining a terminally ill condition in Taiwan. Different countries adopt varied models for providing end-of-life care. In many countries with well-established palliative care consultative services in acute care settings, palliative care teams often manage many aspects of end-of-life care, including communication, ethical reflection, and clinical decision-making. In such contexts, the perceived need for additional ethics consultations may be reduced. In our institution, however, culturally influenced tensions, legal ambiguity, and family dynamics often result in ethically complex situations, particularly concerning the withdrawal of life-sustaining treatments. In these cases, the CEC process offers additional support by facilitating structured communication, clarifying ethical and legal principles, and sharing the burden of difficult decisions with the clinical team (Tapper 2013). While our findings support the utility of mandatory CEC in enhancing the consistency and quality of end-of-life care, we recognize that this model might represent a tailored response to the unique challenges within Taiwan’s healthcare culture. Future comparative studies are warranted to examine the effectiveness, adaptability, and cultural relevance of different ethics consultation models across diverse health systems.

The experiences of CEC and clinical teams significantly impact end-of-life decision-making and patient outcomes. Research has established a volume–outcome relationship, particularly in critical care (Nguyen et al. 2015) and trauma care (Lin et al. 2014). Critically ill patients tend to experience better outcomes when treated in hospitals with higher patient throughput. Following the implementation of a mandatory CEC policy, there has been an increase in the frequency of CEC consultations. As patient volumes rise, both CEC and clinical teams are enhancing their competencies in assessing and communicating effectively with patients and their families. This accumulation of knowledge and experience is expected to further improve patient outcomes. Moreover, the leader of the CEC team assisted in evaluating whether patients were suffering from terminal conditions, and with the accumulation of many experiences, more precise predictions of prognoses and higher quality of end-of-life care will be provided. Moreover, in cases where patients survive the withdrawal of life-sustaining therapies, the consultation team facilitates the transition to hospice care and maintains ongoing clinical visits, even to the eventual discharge of patients. Our team is responsible for providing ethics consultation while delivering comprehensive, holistic, and patient-centred care tailored to individual needs.

It is challenging and difficult to predict the prognosis for patients with neurologic disorders (Bongiovanni et al. 2020; Maciel et al. 2020; Wijdicks 2020). Some authors reported that one of the factors associated with the withdrawal of a ventilator was the physician’s prediction that the patient’s future cognitive function would be severely impaired (Cook et al. 2003). One study revealed significant unexplained variation in the odds of withdrawal of life-sustaining therapy for perceived poor neurologic prognosis across participating hospitals (Steinberg et al. 2021). Another study reported that physicians varied in prognostic estimates and treatment recommendations for patients with intracerebral haemorrhage (Zahuranec et al. 2016). When neurologic patients are at the end of life, the decision to withhold or withdraw life-sustaining treatment is challenging and often stressful for healthcare providers. If the decision is made too early, it jeopardizes patient survival. However, if the timing of the decision is delayed, it could cause unnecessary pain and suffering for patients and their families. In our study, neurologic patients had significantly higher incidence rates of ethical conflicts than non-neurologic patients. The relatively low mortality rate in hospitals after the withdrawal of life-sustaining treatment for neurologic patients could also reflect this dilemma to some extent. Moreover, for all the years covered by our study, there were significantly more days of ethics consultation to withdraw life-sustaining treatment for neurologic patients than for non-neurologic patients. This could indicate difficulties in reaching a consensus decision regarding treatment plans for patients with neurologic conditions. However, several authors have proposed prognostic models for predicting thirty-day mortality rates among ICU patients with ischemic stroke and those with intracerebral haemorrhage which show promising results (van Valburg et al. 2024). Future studies are recommended to develop and validate prognostic tools for patients with neurologic disorders.

One study reported that neurologic patients who died after the withdrawal of life-sustaining treatment had a significantly shorter hospital LOS than other decedents. The authors explained that this demonstrated indirect evidence that decisions and subsequent deaths might often be made before reliable prognostic data are available (Steinberg et al. 2021). This reflected the unique characteristics of patients with neurologic disorders, for which the prediction of prognoses remains challenging. Our results revealed that the main predictive factor for resource usage and ethical conflicts in neurologic patients was the GCS score. A low GCS score predicted a shorter LOS and lower incidence rates of ethical conflicts. In our study, the ICU and hospital LOS were not shorter for neurologic patients. The involvement of the CEC likely facilitated effective end-of-life decision-making by enhancing communication, mitigating potential personal biases, and fostering a consensus among all parties involved.

A variety of studies have emphasized the significance of fostering an ethical environment and establishing trust in delivering the highest quality of care (Nelson 1997; Lin et al. 2013; Lin et al. 2018; Lin et al. 2022). We contended that the observed reductions in the LOS and resource utilization, in addition to the ethical dilemmas faced by both neurologic and non-neurologic patients, reflected the efficacy of our CEC service and the implementation of a mandatory policy. The enforcement of a mandatory CEC policy has the potential to mitigate any bias associated with individual healthcare providers, establish a uniform standard of care, and enhance the support provided to clinical teams. Additionally, it is imperative to prioritize the education and training of healthcare providers to develop competencies for end-of-life care and foster ethical sensitivity in the ICU. This emphasis is vital for improving patient satisfaction and ensuring that quality end-of-life care is provided to patients. Consequently, the education and training of healthcare providers are of paramount importance. Institutions should prioritize the development of specialized training and educational programmes tailored to the unique characteristics of the patients receiving end-of-life care.

This study has several strengths. First, it represents the first investigation of the comprehensive and longitudinal effects of implementing a mandatory CEC policy for both neurologic and non-neurologic patients. Furthermore, this study identifies a range of critical factors that will aid clinical and consultation teams in developing future educational and training programmes tailored to the diverse characteristics of patients concerning end-of-life care.

Our study also has limitations that should be acknowledged. This study was limited to a single institution and a specific context involving patients who were undergoing withdrawal of life-sustaining treatment. Consequently, these findings may not be generalizable to other institutions or scenarios. Although our CEC model and mandatory policy reveal promising results and high satisfaction for family members, future studies will be needed to investigate how ethically optimal consensus might best be reached and how it will align with patient-centred goals, particularly concerning neurologically impaired populations where prognostic uncertainty and potential physician bias might influence outcomes. Though the team leader accumulated many experiences by participating in the CEC process to assist in evaluating whether patients were suffering from terminal conditions, it is possible that bias might still influence future evaluations. Accordingly, two specialists evaluated terminal conditions in order that objectivity would be ensured. Ethical conflicts might be different in different countries and cultures. In our study, the ethical contexts were identified based on value-laden conflicts. Future studies are needed to explore the outcomes of CEC in different cultural environments. Though our results revealed that family members were highly satisfied, potential biases might exist due to sampling method and response rate. The timely assessment of the experiences of bereaved family members presents a significant challenge in maintaining a proper balance during the informed consent process within CEC support. This challenge might have contributed to misunderstanding regarding the distinction between care and evaluation for the participants, potentially influencing the results obtained in this context. Moreover, though our CEC policy revealed promising findings, it should necessarily be validated in a separate control group to establish a causal relationship with the observed outcomes.

An issue not addressed by our study is whether the wishes of patients regarding withdrawal of life-sustaining treatments might contribute to end-of-life decision-making. One study reported that, among older trauma patients, those with an advance directive limiting care underwent withdrawal of life-sustaining support more often than those without it (Badrinathan et al. 2023). Further research might explore this relationship. Furthermore, in the context of our study, the process of designating a surrogate decision-maker in the absence of an advance directive is governed by national legislation. It is acknowledged that surrogate decision-making can be subject to potential biases, including emotional distress, family conflicts, or personal values that may differ from the patient’s presumed wishes. These dynamics may have an influence on how decisions are made, especially in complex, ethically challenging situations. Nevertheless, the CEC does not have legal authority to override the surrogate’s final decision unless the decision clearly violates legal or ethical standards. In such cases, institutional mechanisms, such as additional consultation with legal counsel or hospital ethics committees, may be initiated. Future studies could explore the extent to which such biases might affect end-of-life decisions, and investigate strategies to support more patient-centred surrogate decision-making. Training programmes for healthcare providers must be planned meticulously to ensure their effectiveness. Future research should investigate the educational outcomes associated with these programmes. Some authors illustrated that the implementation of mandatory ethics consultation policies was successful by reporting increases in the number of consultations (Romano et al. 2009). Our results revealed further promising consequences, with decreased resource usage and ethical conflicts as well as higher family member satisfaction. In spite of this, several issues require further exploration, including the appropriateness of mandatory CEC policies and how to maintain CEC models in a variety of situations and cultural environments. Future studies will need to investigate these issues.

## Conclusion

In local healthcare contexts, secular trends in resource usage and ethical conflicts decreased in both neurologic and non-neurologic patients after the implementation of mandatory CEC. Neurologic patients showed a greater decrease in resource usage than non-neurologic patients. Neurologic patients had significantly higher incidence rates of ethical conflict than non-neurologic patients. The main predictive factor for resource usage and ethical conflicts in neurologic patients was the GCS score. Our results provide CEC and clinical teams with knowledge that will better equip them to approach the distinct issues raised by various patient characteristics. Institutions should consider the potential value of implementing mandatory CEC policies and developing special educational and training programmes to provide the best quality of end-of-life care in ICUs, though the generalizability of these results to other healthcare systems requires cautious interpretation.

## Supplementary Information

Below is the link to the electronic supplementary material.

Supplementary file1 (DOCX 60 KB)

## Data Availability

The datasets generated and/or analysed during the current study are not publicly available but are available from the corresponding author on reasonable request.

## Declarations

We confirm that this manuscript complies with all instructions to authors.

The final manuscript was approved by all authors.

This manuscript has not been published elsewhere and is not under consideration by another journal.

The reporting checklist, STROBE statement, was used and uploaded with the submission.
