# Correction to: Impact of Mandatory Clinical Ethics Consultation for Neurocritically Ill Patients

**DOI:** 10.1007/s11673-026-10595-z

**Published:** 2026-06-09

**Authors:** Yen-Ko Lin, Chao-Wen Chen, Yung-Sung Yeh, Chia-Ju Lin, Yu-Wen Huang, Yu-Chih Lin, Chau-Chyun Sheu

**Affiliations:** 1 Division of Trauma and Surgical Critical Care, Department of Surgery, Kaohsiung Medical University Hospital, Kaohsiung Medical University, No.100, Tzyou 1st Rd., Sanmin District, Kaohsiung City, 807378 Taiwan; 2 Department of Emergency Medicine, Kaohsiung Medical University Hospital, Kaohsiung Medical University, No.100, Tzyou 1st Rd., Sanmin District, Kaohsiung City, 807378 Taiwan; 3 Department of Medical Humanities and Education, College of Medicine, Kaohsiung Medical University, No.100, Shiquan 1st Rd., Sanmin District, Kaohsiung City, 807378 Taiwan; 4 Center for Medical Education and Humanizing Health Professional Education, Kaohsiung Medical University, No.100, Shiquan 1st Rd., Sanmin District, Kaohsiung City, 807378 Taiwan; 5 Department of Emergency Medicine, College of Medicine, Kaohsiung Medical University, No.100, Shiquan 1st Rd., Sanmin District, Kaohsiung City, 807378 Taiwan; 6 College of Nursing, Kaohsiung Medical University, No. 100, Shiquan 1st Rd., Sanmin District, Kaohsiung City, 807378 Taiwan; 7 Department of Nursing, Kaohsiung Medical University Hospital, Kaohsiung Medical University, No. 100, Tzyou 1st Rd., Sanmin District, Kaohsiung City, 807378 Taiwan; 8 Division of Rheumatology, Department of Internal Medicine, College of Medicine, Kaohsiung Medical University Hospital, Kaohsiung Medical University, No. 100, Tzyou 1st Rd., Sanmin District, Kaohsiung City, 807378 Taiwan; 9 Department of Internal Medicine, College of Medicine, Kaohsiung Medical University, No. 100, Shiquan 1st Rd., Sanmin District, Kaohsiung City, 807378 Taiwan; 10 Division of Pulmonary and Critical Care Medicine, Department of Internal Medicine, Kaohsiung Medical University Hospital, Kaohsiung Medical University, No. 100, Tzyou 1st Rd., Sanmin District, Kaohsiung City, 807378 Taiwan

**Correction to: Journal of Bioethical Inquiry**

**https://doi.org/10.1007/s11673-025-10512-w**

In this article, the authors' surnames were correct, but their first names were abbreviated. Their full first names should have been published instead of the initials.

The original article has been corrected.

