# Supplementary material for: Impact of Mandatory Clinical Ethics Consultation for Neurocritically Ill Patients
Source: J Bioeth Inq. 2026 May 28;23(3):643–55. doi: 10.1007/s11673-025-10512-w (PMC13645955; doi:10.1007/s11673-025-10512-w)
Supplement: Supplementary file 1 [file 11673_2025_10512_MOESM1_ESM.docx]

Supplementary Information

**Impact of Mandatory Clinical Ethics Consultation for Neurocritically Ill Patients**

| Table 1. Baseline characteristics of neurologic and non-neurologic patients | | | | |
| --- | --- | --- | --- | --- |
| Characteristics |  | Neurologic patients (n=437) | Non-neurologic patients (n=713) | *p*-value |
| Age (years) |  | Mean, 63.52; SD, 16.44 | Mean, 68.36; SD, 14.66 | 0.0000 |
|  |  | Median, 66; IQR, 53-76 | Median, 69; IQR, 59-80 |  |
|  |  | number (%) | number (%) |  |
| Male |  | 260 (59.5) | 431 (60.5) | 0.757^a^ |
| Married |  | 268 (61.3) | 452 (63.4) | 0.490^a^ |
| Admission year |  |  |  |  |
| 2013-2014 |  | 54 (12.4) | 83 (11.6) | 0.234^a^ |
| 2015-2017 |  | 181 (41.4) | 264 (37.0) |  |
| 2018-2020 |  | 202 (46.2) | 366 (51.3) |  |
| Surrogate |  |  |  | 0.030^a^ |
| Spouse |  | 87 (19.9) | 125 (17.5) |  |
| Offspring |  | 265 (60.6) | 471 (66.1) |  |
| Parents |  | 30 (6.9) | 28 (3.9) |  |
| Sibling |  | 46 (10.5) | 61 (8.6) |  |
| Others |  | 9 (2.1) | 28 (3.9) |  |
| Expression of wish |  | 78 (17.9) | 176 (24.7) | 0.007^a^ |
| Advance directive |  | 16 (3.7) | 34 (4.8) | 0.457^a^ |
| Cardiac arrest |  | 77 (17.6) | 220 (30.9) | 0.000^a^ |
| Inotropes or vasopressors |  | 67 (15.3) | 264 (37.0) | 0.000^a^ |
| Died in hospital |  | 328 (75.1) | 674 (94.5) | 0.000^a^ |
| GCS score |  | Mean, 4.60; SD, 1.95 | Mean, 5.31; SD, 2.68 | 0.002 |
|  |  | Median, 4; IQR, 3-6 | Median, 4; IQR, 3-7 |  |

^a^ Fisher’s exact test

Abbreviation: SD, standardized deviation; IQR, interquartile range; GCS: Glasgow coma scale

Table 2. Secular trends and comparison of neurologic and non-neurologic patients for outcomes

| **characteristic** | All years | |  | 2013-2014 | |  | 2015-2017 | |  | 2018-2020 | |  | *p*-value for trends | |
| --- | --- | --- | --- | --- | --- | --- | --- | --- | --- | --- | --- | --- | --- | --- |
|  | Neurologic patients | Non-neurologic patients | *p*-value | Neurologic patients | Non-neurologic patients | *p*-value | Neurologic patients | Non-neurologic patients | *p*-value | Neurologic patients | Non-neurologic patients | *p*-value | Neurologic patients | Non-neurologic patients |
| **No. of patients** | 437 | 713 |  | 54 | 83 |  | 181 | 264 |  | 202 | 366 |  |  |  |
| **ICU length of stay, day** |  |  |  |  |  |  |  |  |  |  |  |  | 0.004 | 0.014 |
| Mean (SD) | 17.49 (12.48) | 17.20 (13.23) | 0.432 | 18.17 (11.26) | 19.53 (13.05) | 0.573 | 18.29 (10.97) | 17.17 (12.76) | 0.047 | 16.59 (13.97) | 16.70 (13.59) | 0.475 |  |  |
| Median (IQR) | 15 (9-22) | 15 (8-22) |  | 17 (11-22) | 18 (10-25) |  | 16 (11-23) | 14 (9-22) |  | 12 (7-22) | 14 (8-20) |  |  |  |
| **Total length of stay, day** |  |  |  |  |  |  |  |  |  |  |  |  | 0.0001 | 0.082 |
| Mean (SD) | 26.28 (21.11) | 25.43 (21.12) | 0.906 | 30.07 (19.30) | 27.12 (19.12) | 0.287 | 28.79 (20.90) | 26.63 (23.78) | 0.129 | 23.02 (21.38) | 24.19 (19.42) | 0.054 |  |  |
| Median (IQR) | 20 (10-38) | 20 (11-34) |  | 28 (13-42) | 23 (13-34) |  | 24 (12-39) | 21 (11-36) |  | 16 (8-32) | 19 (10-33) |  |  |  |
| **Ventilator use, day** |  |  |  |  |  |  |  |  |  |  |  |  | 0.000 | 0.001 |
| No. of patients | 433 | 704 |  | 54 | 83 |  | 179 | 260 |  | 200 | 361 |  |  |  |
| Mean (SD) | 18.39 (14.24) | 17.88 (17.31) | 0.302 | 22.04 (16.01) | 19.53 (13.90) | 0.502 | 21.03 (14.26) | 19.69 (20.24) | 0.021 | 15.04 (12.99) | 16.19 (15.50) | 0.179 |  |  |
| Median (IQR) | 14 (8-26) | 14 (8-22) |  | 18 (11-33) | 16 (10-24) |  | 16 (10-28) | 14 (8-25.5) |  | 10 (6-19) | 13 (7-19) |  |  |  |
| **ICU admission to ethics consultation, day** |  |  |  |  |  |  |  |  |  |  |  |  | 0.000 | 0.0092 |
| Mean (SD) | 14.06 (13.41) | 15.36 (15.83) | 0.241 | 17.02 (14.37) | 16.92 (14.49) | 0.970 | 15.39 (13.06) | 16.69 (18.61) | 0.686 | 12.08 (13.22) | 14.05 (13.72) | 0.014 |  |  |
| Median (IQR) | 9 (5-19) | 11 (6-20) |  | 11 (7-22) | 13 (7-21) |  | 12 (8-21) | 12 (6-21.5) |  | 7 (4-14) | 10 (5-18) |  |  |  |
| **Ethics consultation to withdrawal of life-sustaining treatment, day** |  |  |  |  |  |  |  |  |  |  |  |  | 0.0003 | 0.033 |
| Mean (SD) | 3.47 (4.62) | 2.16 (3.53) | 0.000 | 4.98 (6.30) | 3.63 (4.44) | 0.232 | 4.00 (5.05) | 1.83 (3.34) | 0.000 | 2.58 (3.38) | 2.06 (3.36) | 0.083 |  |  |
| Median (IQR) | 2 (1-4) | 1 (0-3) |  | 3 (1-5) | 2 (1-5) |  | 2 (1-5) | 1 (0-2) |  | 1 (0-3) | 1 (0-3) |  |  |  |

Table 3. Subgroup analysis for ICU LOS, total LOS, and the days of ventilator use between neurologic and non-neurologic patients

|  |  | ICU length of stay | | | | |  | | Total length of stay | | | | |  | Ventilator use, day | | | | | | | | | | |  |
| --- | --- | --- | --- | --- | --- | --- | --- | --- | --- | --- | --- | --- | --- | --- | --- | --- | --- | --- | --- | --- | --- | --- | --- | --- | --- | --- |
| Variable |  | Neurologic patients | | Non-neurologic patients | | | *p*-value |  | Neurologic patients | | Non-neurologic patients | | | *p*-  value | |  | Neurologic patients | | Non-neurologic patients | | | | | *p*-value | |  |
|  | n | Mean (SD) | Median (IQR) | n | Mean (SD) | Median (IQR) |  | n | Mean (SD) | Median (IQR) | n | Mean (SD) | Median (IQR) |  | | n | Mean (SD) | Median (IQR) | n | | Mean (SD) | | Median (IQR) | |  | |
| Age (years) |  |  |  |  |  |  |  |  |  |  |  |  |  |  | |  |  |  |  |  | |  | |  | |  |
| <68 | 238 | 17.71 (13.19) | 14  (9-24) | 321 | 16.82 (14.85) | 13  (7-21) | 0.159 | 238 | 26.44 (22.08) | 18.5  (9-38) | 321 | 25.78 (21.41) | 20  (11-34) | 0.875 | | 236 | 18.60 (14.91) | 14  (8-26) | 315 | | 17.50 (17.47) | 13  (7-21) | | 0.170 | |  |
| ≥68 | 199 | 17.22 (11.60) | 16  (9-22) | 392 | 17.52 (11.76) | 15  (9-23) | 0.907 | 199 | 26.10 (19.94) | 20  (10-37) | 392 | 25.16 (20.91) | 20  (11-34) | 0.708 | | 197 | 18.14 (13.43) | 13  (8-25) | 389 | | 18.19 (17.19) | 14  (8-23) | | 0.843 | |  |
| Gender |  |  |  |  |  |  |  |  |  |  |  |  |  |  | |  |  |  |  | |  |  | |  | |  |
| Female | 177 | 17.75  (13.52) | 15  (9-22) | 282 | 16.13  (12.96) | 13  (8-21) | 0.117 | 177 | 26.28  (20.95) | 20  (10-37) | 282 | 24.42  (23.84) | 16  (9-34) | 0.197 | | 175 | 18.54  (14.72) | 13  (8-28) | 278 | | 17.08  (19.06) | 12  (7-20) | | 0.093 | |  |
| Male | 260 | 17.31  (11.74) | 15  (9-23) | 431 | 17.90  (13.38) | 15  (9-23) | 0.811 | 260 | 26.28  (21.26) | 20  (9-38) | 431 | 26.10 (19.13) | 21  (12-34) | 0.384 | | 258 | 18.29  (13.93) | 14  (8-25) | 426 | | 18.40  (16.06) | 15  (8-23) | | 0.989 | |  |
| Current marital status |  |  |  |  |  |  |  |  |  |  |  |  |  |  | |  |  |  |  | |  |  | |  | |  |
| Married | 268 | 17.01  (11.86) | 15  (9-22) | 452 | 17.54  (13.52) | 15  (8.5-22) | 0.987 | 268 | 25.32  (19.72) | 19  (9.5-37) | 452 | 25.47  (19.20) | 21  (11.5-33) | 0.531 | | 266 | 18.81  (14.10) | 14  (9-27) | 447 | | 18.27  (16.10) | 14  (8-23) | | 0.389 | |  |
| Others | 169 | 18.24  (13.41) | 15  (9-24) | 261 | 16.62  (12.73) | 14  (8-21) | 0.215 | 169 | 27.80  (23.12) | 21  (10-38) | 261 | 25.38  (24.12) | 19  (10-36) | 0.333 | | 167 | 17.72  (14.47) | 12  (8-23) | 257 | | 17.21  (19.24) | 13  (7-20) | | 0.517 | |  |
| Advance cancer |  |  |  |  |  |  |  |  |  |  |  |  |  |  | |  |  |  |  | |  |  | |  | |  |
| Yes | 59 | 14.58  (10.70) | 12  (7-18) | 269 | 16.46  (10.50) | 14  (8-20) | 0.240 | 59 | 23.75  (19.63) | 18  (11-30) | 269 | 26.65  (19.94) | 218  (12-36) | 0.196 | | 57 | 15.89  (12.31) | 13  (6-25) | 266 | | 16.97  (13.18) | 13  (8-20) | | 0.457 | |  |
| No | 378 | 17.94  (12.69) | 15.5  (9-22) | 444 | 17.65  (13.90) | 15  (8-23) | 0.421 | 378 | 26.68  (21.33) | 20  (9-38) | 444 | 24.70  (21.79) | 19  (10-33) | 0.290 | | 367 | 18.77  (14.49) | 14  (9-26) | 438 | | 18.43  (19.38) | 14  (8-23) | | 0.250 | |  |
| Cardiac arrest |  |  |  |  |  |  |  |  |  |  |  |  |  |  | |  |  |  |  | |  |  | |  | |  |
| Yes | 77 | 16.56  (14.19) | 12  (8-20) | 220 | 14.40  (13.06) | 11  (7-18) | 0.118 | 77 | 24.06  (24.07) | 13  (9-33) | 220 | 18.13  (16.27) | 13  (7-23.5) | 0.174 | | 77 | 17.62  (16.03) | 11  (8-20) | 220 | | 15.56  (17.70) | 10  (6.5-18.5) | | 0.141 | |  |
| No | 360 | 17.69  (12.10) | 16  (9-22.5) | 493 | 18.45  (13.13) | 16  (10-23) | 0.476 | 360 | 26.76  (20.43) | 21  (10-38) | 493 | 28.70  (22.21) | 23  (14-38) | 0.080 | | 356 | 18.55  (13.84) | 14  (8.5-26) | 484 | | 18.93  (17.04) | 15  (8-23) | | 0.761 | |  |
| Inotropes or vasopressors |  |  |  |  |  |  |  |  |  |  |  |  |  |  | |  |  |  |  | |  |  | |  | |  |
| Yes | 67 | 18.76  (17.37) | 11  (7-26) | 264 | 15.81  (12.51) | 13  (7-21) | 0.511 | 67 | 25.45  (25.45) | 14  (7-36) | 264 | 23.56  (20.44) | 19  (9-32.5) | 0.565 | | 67 | 18.84  (18.27) | 11  (6-26) | 263 | | 15.97  (16.51) | 12  (7-20) | | 0.454 | |  |
| No | 370 | 17.26  (11.39) | 15  (9-22) | 449 | 18.02  (13.59) | 15  (9-22) | 0.873 | 370 | 26.43  (20.26) | 20.5  (10-38) | 449 | 26.53  (21.46) | 21  (12-34) | 0.703 | | 366 | 18.31  (13.40) | 14  (9-25) | 441 | | 19.02  (17.68) | 15  (9-23) | | 0.768 | |  |
| GCS score |  |  |  |  |  |  |  |  |  |  |  |  |  |  | |  |  |  |  | |  |  | |  | |  |
| ≤4 | 264 | 15.33  (11.83) | 12  (7-21) | 369 | 15.88  (13.21) | 13  (7-20) | 0.946 | 264 | 22.26  (20.95) | 14  (8-30) | 369 | 22.27  (21.92) | 16  (9-29) | 0.481 | | 261 | 16.28  (13.66) | 11  (7-23) | 369 | | 16.99  (19.45) | 12  (7-21) | | 0.891 | |  |
| >4 | 173 | 20.78  (12.77) | 18  (12-24) | 344 | 18.63  (13.13) | 16  (10-23) | 0.025 | 173 | 32.42  (19.89) | 29  (17-44) | 344 | 28.83  (19.70) | 24  (14-38.5) | 0.029 | | 172 | 21.58  (14.54) | 18  (10-28) | 335 | | 18.86  (14.55) | 15  (9-23) | | 0.018 | |  |

Table 4. Multiple linear regression model for ICU length of stay, total length of stay, and ventilator use

|  | ICU length of stay^a^ | | | | Total length of stay^a^ | | | | | Ventilator use, day^b^ | | | |
| --- | --- | --- | --- | --- | --- | --- | --- | --- | --- | --- | --- | --- | --- |
|  | Neurologic patients | | Non-neurologic patients | | | Neurologic patients | | Non-neurologic patients | | Neurologic patients | | Non-neurologic patients | |
|  | Coefficient | 95%CI | Coefficient | 95%CI | | Coefficient | 95%CI | Coefficient | 95%CI | Coefficient | 95%CI | Coefficient | 95%CI |
| **Age, y** | -0.071 | -0.143-0.002 | 0.039 | -0.029-0.106 | | -0.155* | -0.276--0.034 | -0.014 | -0.120-0.093 | -0.063 | -0.146-0.021 | 0.014 | -0.077-0.104 |
| **Gender**  (reference group=male) | 0.781 | -1.567-3.130 | -1.794 | -3.789-0.202 | | 0.496 | -3.412-4.404 | -1.330 | -4.476-1.815 | 0.591 | -2.108-3.290 | -1.180 | -3.836-1.476 |
| **Current marital status** | -0.690 | -3.074-1.694 | 0.879 | -1.160-2.918 | | -1.569 | -5.536-2.398 | 0.450 | -2.763-3.663 | 1.513 | -1.229-4.9256 | 1.200 | -1.512-3.913 |
| (reference group=others) |  | |  | | |  | |  | |  | |  | |
| **Advanced cancer**  (reference group=no cancer) | -3.570* | -6.922--0.218 | -2.055* | -4.090--0.020 | | -3.319 | -8.898-2.260 | -0.221 | -3.429-2.986 | -3.172 | -7.067-0.722 | -2.268 | -4.971-0.435 |
| **Cardiac arrest**  (reference group=no cardiac arrest) | -0.391 | -3.478-2.697 | -4.289*** | -6.513--2.065 | | -0.236 | -5.374-4.902 | -9.530*** | -13.035--6.024 | -0.011 | -3.543-3.521 | -3.538* | -6.476--0.600 |
| **Inotropes or vasopressors**  (reference group=no inotropes or vasopressors) | 2.722 | -0.553-5.997 | -1.876 | -3.894-0.142 | | 1.408 | -4.042-6.857 | -2.023 | -5.204-1.158 | 2.097 | -1.647-5.841 | -2.779* | -5.450--0.107 |
| **GCS score** | 1.567*** | 0.596-2.177 | 0.059 | -0.327-0.444 | | 3.389*** | 2.373-4.404 | 0.538 | -0.070-1.146 | 1.882*** | 1.178-2.585 | 0.119 | -0.419-0.656 |
| **Constant** | 14.219*** | 8.170-20.267 | 18.989*** | 13.229-24.748 | | 21.058*** | 10.991-31.123 | 28.851*** | 19.772-37.929 | 12.044*** | 5.118-18.969 | 20.216*** | 12.586-27.845 |
|  | R squared = 0.072 |  | R squared = 0.038 |  | | R squared = 0.102 |  | R squared = 0.062 |  | R squared = 0.069 |  | R squared = 0.021 |  |

**p*< 0.05; ***p* < 0.01; ****p* < 0.001. ^a^Sample size of regression model = 1150; ^b^Sample size of regression model = 1137.

Table 5. Secular trends and comparison of incidence rate of ethical contexts for neurologic and non-neurologic patients

| **characteristic** | All years | |  | 2013-2014 | |  | 2015-2017 | |  | 2018-2020 | |  | *p*-value for trends | |
| --- | --- | --- | --- | --- | --- | --- | --- | --- | --- | --- | --- | --- | --- | --- |
|  | Neurologic patients | Non-neurologic patients | *p*-value | Neurologic patients | Non-neurologic patients | *p*-value | Neurologic patients | Non-neurologic patients | *p*-value | Neurologic patients | Non-neurologic patients | *p*-value | Neurologic patients | Non-neurologic patients |
|  | n (%) | n (%) |  | n (%) | n (%) |  | n (%) | n (%) |  | n (%) | n (%) |  |  |  |
| **Conflicts concerning determining terminally ill condition** | 62 (14.2) | 75 (10.5) | 0.074 | 16 (29.6) | 21 (25.3) | 0.694 | 20 (11.1) | 11 (4.2) | 0.007 | 26 (12.9) | 43 (11.8) | 0.690 | 0.028 | 0.168 |
| **Conflicts between family members** | 257 (58.8) | 353 (49.5) | 0.002 | 32 (59.3) | 45 (54.2) | 0.600 | 115 (63.5) | 130 (49.2) | 0.004 | 110 (54.5) | 178 (48.6) | 0.190 | 0.201 | 0.438 |
| **Conflicts between family members and clinical team** | 225 (51.5) | 267 (37.5) | 0.000 | 32 (59.3) | 37 (44.6) | 0.116 | 112 (61.9) | 118 (44.7) | 0.000 | 81 (40.1) | 112 (30.6) | 0.026 | 0.0001 | 0.0005 |
| **Conflicts between clinical team members** | 72 (16.5) | 68 (9.5) | 0.001 | 11 (20.4) | 13 (15.7) | 0.498 | 39 (21.6) | 28 (10.6) | 0.002 | 22 (10.9) | 27 (7.4) | 0.162 | 0.014 | 0.016 |

Table 6. Subgroup analysis of incidence rate of ethical contexts for neurologic and non-neurologic patients

|  | Conflicts concerning determining terminally ill condition | | | Conflicts between family members | | | Conflicts between family members and clinical team | | | Conflicts between clinical team members | | |
| --- | --- | --- | --- | --- | --- | --- | --- | --- | --- | --- | --- | --- |
|  | Neurologic patients | Non-neurologic patients | *p*-value | Neurologic patients | Non-neurologic patients | *p*-value | Neurologic patients | Non-neurologic patients | *p*-value | Neurologic patients | Non-neurologic patients | *p*-value |
|  | n (%) | n (%) |  | n (%) | n (%) |  | n (%) | n (%) |  | n (%) | n (%) |  |
| **Age** |  |  |  |  |  |  |  |  |  |  |  |  |
| <68 | 32 (13.5) | 34 (10.6) | 0.354 | 143 (60.1) | 172 (53.6) | 0.143 | 130 (54.6) | 130 (40.5) | 0.001 | 46 (19.3) | 34 (10.6) | 0.005 |
| ≥68 | 30 (15.1) | 41 (10.5) | 0.109 | 114 (57.3) | 181 (46.2) | 0.012 | 95 (47.7) | 137 (34.9) | 0.003 | 26 (13.1) | 34 (8.7) | 0.113 |
| **Gender** |  |  |  |  |  |  |  |  |  |  |  |  |
| Female | 19 (10.7) | 31 (11.0) | 1.000 | 103 (58.2) | 123 (43.6) | 0.003 | 91 (51.4) | 99 (35.1) | 0.001 | 23 (13.0) | 24 (8.5) | 0.154 |
| Male | 43 (16.5) | 44 (10.2) | 0.018 | 154 (59.2) | 230 (53.4) | 0.134 | 134 (51.5) | 168 (39.0) | 0.002 | 49 (18.9) | 44 (10.2) | 0.002 |
| **Current marital status** |  |  |  |  |  |  |  |  |  |  |  |  |
| Married | 36 (13.4) | 40 (8.9) | 0.060 | 145 (54.1) | 202 (44.7) | 0.017 | 127 (47.4) | 154 (34.1) | 0.000 | 35 (13.1) | 38 (8.4) | 0.055 |
| Others | 26 (15.4) | 35 (13.4) | 0.574 | 112 (66.3) | 151 (57.9) | 0.086 | 98 (58.0) | 113 (43.3) | 0.003 | 37 (21.9) | 30 (11.5) | 0.004 |
| **Advanced cancer** |  |  |  |  |  |  |  |  |  |  |  |  |
| Yes | 11 (18.6) | 25 (9.3) | 0.062 | 32 (54.2) | 127 (47.2) | 0.388 | 31 (52.1) | 91 (33.8) | 0.011 | 8 (13.6) | 21 (7.8) | 0.202 |
| No | 51 (13.5) | 50 (11.3) | 0.339 | 225 (59.5) | 226 (50.9) | 0.014 | 194 (51.3) | 176 (39.6) | 0.001 | 64 (16.9) | 47 (10.6) | 0.010 |
| **Cardiac arrest** |  |  |  |  |  |  |  |  |  |  |  |  |
| Yes | 6 (7.8) | 20 (9.1) | 0.819 | 41 (53.3) | 102 (46.4) | 0.354 | 37 (48.1) | 75 (34.1) | 0.040 | 13 (16.9) | 21 (9.6) | 0.096 |
| No | 56 (15.6) | 55 (11.2) | 0.064 | 216 (60.0) | 251 (50.9) | 0.010 | 188 (52.2) | 192 (38.9) | 0.000 | 59 (16.4) | 47 (9.5) | 0.003 |
| **Inotropes or vasopressors** |  |  |  |  |  |  |  |  |  |  |  |  |
| Yes | 6 (9.0) | 20 (7.6) | 0.799 | 40 (59.7) | 128 (48.5) | 0.132 | 27 (40.3) | 87 (32.9) | 0.314 | 11 (16.4) | 15 (5.7) | 0.008 |
| No | 56 (15.1) | 55 (12.3) | 0.259 | 217 (58.7) | 225 (50.1) | 0.017 | 198 (53.5) | 180 (40.1) | 0.000 | 61 (16.5) | 53 (11.8) | 0.068 |
| **GCS score** |  |  |  |  |  |  |  |  |  |  |  |  |
| ≤4 | 18 (6.8) | 31 (8.4) | 0.547 | 138 (52.3) | 160 (43.4) | 0.029 | 121 (45.8) | 111 (30.1) | 0.000 | 28 (10.6) | 29 (7.9) | 0.261 |
| >4 | 44 (25.4) | 44 (12.8) | 0.000 | 119 (68.8) | 193 (56.1) | 0.006 | 104 (60.1) | 156 (45.4) | 0.002 | 44 (25.4) | 39 (11.3) | 0.000 |

Table 7. Multivariable logistic regression models of incidence rate of ethical contexts

|  | Conflicts concerning determining terminally ill condition | | |  | Conflicts between family members | | |  | Conflicts between family members and clinical team | | |  | Conflicts between clinical team members | | |  |
| --- | --- | --- | --- | --- | --- | --- | --- | --- | --- | --- | --- | --- | --- | --- | --- | --- |
|  | Neurologic patients | | Non-neurologic patients | | Neurologic patients | | Non-neurologic patients | | Neurologic patients | | Non-neurologic patients | | Neurologic patients | | Non-neurologic patients | |
|  | Odds ratio | 95% CI | Odds ratio | 95% CI | Odds ratio | 95% CI | Odds ratio | 95% CI | Odds ratio | 95% CI | Odds ratio | 95% CI | Odds ratio | 95% CI | Odds ratio | 95% CI |
| **Age, y** | 0.997 | 0.980-1.015 | 0.996 | 0.980-1.012 | 0.995 | 0.982-1.008 | 0.988* | 0.977-0.999 | 0.992 | 0.980-1.005 | 0.993 | 0.983-1.004 | 0.979** | 0.964-0.995 | 0.995 | 0.979-1.013 |
| **Gender**  (reference group=male) | 0.559 | 0.304-1.029 | 1.087 | 0.659-1.793 | 0.969 | 0.649-1.448 | 0.661** | 0.482-0.906 | 0.990 | 0.668-1.466 | 0.846 | 0.612-1.170 | 0.691 | 0.396-1.208 | 0.831 | 0.485-1.424 |
| **Current marital status**  (reference group=others) | 0.826 | 0.460-1.481 | 0.674 | 0.410-1.070 | 0.618* | 0.410-0.933 | 0.596** | 0.432-0.822 | 0.665* | 0.446-0.992 | 0.707* | 0.510-0.980 | 0.589 | 0.344-1.010 | 0.745 | 0.440-1.261 |
| **Advanced cancer**  (reference group=no cancer) | 1.412 | 0.660-3.019 | 0.768 | 0.455-1.296 | 0.798 | 0.452-1.411 | 0.780 | 0.566-1.076 | 1.068 | 0.610-1.871 | 0.711* | 0.510-0.991 | 0.773 | 0.341-1.752 | 0.681 | 0.389-1.191 |
| **Cardiac arrest**  (reference group=no cardiac arrest) | 0.654 | 0.260-1.643 | 0.940 | 0.524-1.687 | 0.888 | 0.528-1.494 | 0.994 | 0.701-1.409 | 1.029 | 0.614-1.724 | 0.975 | 0.678-1.400 | 1.232 | 0.607-2.503 | 1.303 | 0.715-2.376 |
| **Inotropes or vasopressors**  (reference group=no inotropes or vasopressors) | 0.758 | 0.298-1.927 | 0.645 | 0.373-1.113 | 1.237 | 0.707-2.163 | 1.073 | 0.781-1.474 | 0.604 | 0.347-1.051 | 0.829 | 0.597-1.152 | 1.003 | 0.472-2.134 | 0.505* | 0.276-0.925 |
| **GCS score** | 1.387*** | 1.216-1.583 | 1.091 | 0.998-1.193 | 1.225*** | 1.093-1.373 | 1.128*** | 1.060-1.201 | 1.113* | 1.003-1.236 | 1.132*** | 1.064-1.204 | 1.245*** | 1.097-1.414 | 1.139** | 1.038-1.249 |
| **Constant** | 0.096** | 0.022-0.411 | 0.140** | 0.035-0.556 | 1.135 | 0.397-3.247 | 3.185* | 1.269-7.991 | 1.461 | 0.529-4.042 | 0.920 | 0.368-2.301 | 0.551 | 0.153-1.984 | 0.136** | 0.032-0.585 |
| Likelihood ratio test for model | *χ2*= 34.11; *P* < 0.000 | | χ2= 12.20; P = 0.094 | | *χ2*=21.14; *P* < 0.0036 | | *χ2*= 40.35; *P* < 0.000 | | *χ2*=13.96; *P* = 0.0519 | | *χ2*= 31.05; *P* = 0.0001 | | *χ2*= 24.34; *P* = 0.0010 | | *χ2*= 18.64; *P* = 0.0094 | |

| Table 8. Baseline characteristics of family members in neurologic and non-neurologic patients | | | | | |
| --- | --- | --- | --- | --- | --- |
| Characteristics |  | Neurologic (n=50) | Non-neurologic (n=62) | *p*-value | |
|  |  | number (%) | number (%) |  | |
| Age |  |  |  | 0.514^a^ | |
| 18-29 |  | 2 (4.0) | 7 (11.3) |  | |
| 30-39 |  | 13 (26.0) | 11 (17.7) |  | |
| 40-49 |  | 9 (18.0) | 15 (24.2) |  | |
| 50-59 |  | 18 (36.0) | 20 (32.3) |  | |
| >59 |  | 8 (16.0) | 9 (14.5) |  | |
| Gender |  |  |  | 0.849^a^ | |
| Female |  | 26 (52.0) | 34 (54.8) |  | |
| Male |  | 24 (48.0) | 28 (45.2) |  |  |
| Education |  |  |  | 0.088^a^ |  |
| <High school |  | 2 (4.0) | 6 (9.7) |  |  |
| High school |  | 26 (52.0) | 20 (32.3) |  |  |
| College and post-graduate |  | 22 (44.0) | 36 (58.1) |  |  |
| Relationship |  |  |  | 0.552^a^ |  |
| Spouse |  | 6 (12.0) | 5 (8.1) |  | |
| Offspring |  | 30 (60.0) | 42 (67.7) |  | |
| Parents |  | 3 (6.0) | 3 (4.8) |  | |
| Sibling |  | 4 (8.0) | 8 (12.9) |  | |
| Others |  | 7 (14.0) | 4 (6.5) |  | |
| ^a^ Fisher’s exact test | | | | | |

| Table 9. Family member satisfaction regarding mandatory clinical ethics consultation. | | | | | | | |
| --- | --- | --- | --- | --- | --- | --- | --- |
| Item No. | | Item | Neurologic (n=67) | | Non-neurologic (n=45) | | *p*-value^a^ |
|  |  | | Mean (SD) | Median (IQR) | Mean (SD) | Median (IQR) |  |
| 1. | I am satisfied with the care of clinical team. | | 4.80 (0.40) | 5 (5-5) | 4.77 (0.42) | 5 (5-5) | 0.742 |
| 2 | I am satisfied with help of clinical ethics consultation team. | | 4.78 (0.42) | 5 (5-5) | 4.76 (0.43) | 5 (5-5) | 0.786 |
| 3 | I am satisfied with the family meeting hosted by clinical ethics consultation team. | | 4.82 (0.39) | 5 (5-5) | 4.71 (0.46) | 5 (4-5) | 0.177 |
| 4 | I feel that the withdrawal of life-sustaining treatment is not to abandon the patient. | | 4.72 (0.45) | 5 (4-5) | 4.66 (0.48) | 5 (4-5) | 0.558 |
| 5 | Clinical ethics consultation will help me make medical decision for my family. | | 4.72 (0.45) | 5 (4-5) | 4.66 (0.54) | 5 (4-5) | 0.685 |
| 6 | I feel that the patient will not suffer during the process of withdrawal of life-sustaining treatment. | | 4.56 (0.73) | 5 (4-5) | 4.52 (0.67) | 5 (4-5) | 0.572 |
| 7 | I feel that all my concerns have been solved. | | 4.72 (0.45) | 5 (4-5) | 4.66 (0.54) | 5 (4-5) | 0.685 |
| 8 | I feel that my value has been respected. | | 4.66 (0.48) | 5 (4-5) | 4.68 (0.57) | 5 (4-5) | 0.618 |
| 9 | I will recommend other patients or family members to request the clinical ethics consultation during end-of-life care. | | 4.78 (0.42) | 5 (5-5) | 4.67 (0.50) | 5 (4-5) | 0.288 |
| 10 | I feel that the clinical ethics consultation must be provided during end-of-life care. | | 4.78 (0.42) | 5 (5-5) | 4.79 (0.41) | 5 (5-5) | 0.895 |
| ^a^ Wilcoxon rank-sum test  Abbreviation: ICU, intensive care unit; SD, standardized deviation; IQR, interquartile range; GCS: Glasgow coma scale | | | | | | | |
